# Identification of genomic indels and structural variations using split reads

**DOI:** 10.1186/1471-2164-12-375

**Published:** 2011-07-25

**Authors:** Zhengdong D Zhang, Jiang Du, Hugo Lam, Alex Abyzov, Alexander E Urban, Michael Snyder, Mark Gerstein

**Affiliations:** 1 Department of Genetics, Albert Einstein College of Medicine, Bronx, NY 10461, USA; 2 Department of Computer Science, Yale University, New Haven, CT 06520, USA; 3 Interdepartmental Program in Computational Biology and Bioinformatics, Yale University, New Haven, CT 06520, USA; 4 Department of Psychiatry and Behavioral Sciences, Stanford University, Stanford, CA 94305, USA; 5 Department of Genetics, Stanford University, Stanford, CA 94305, USA

**Keywords:** insertion, deletion, structure variation, split read, high-throughput sequencing

## Abstract

**Background:**

Recent studies have demonstrated the genetic significance of insertions, deletions, and other more complex structural variants (SVs) in the human population. With the development of the next-generation sequencing technologies, high-throughput surveys of SVs on the whole-genome level have become possible. Here we present split-read identification, calibrated (SRiC), a sequence-based method for SV detection.

**Results:**

We start by mapping each read to the reference genome in standard fashion using gapped alignment. Then to identify SVs, we score each of the many initial mappings with an assessment strategy designed to take into account both sequencing and alignment errors (e.g. scoring more highly events gapped in the center of a read). All current SV calling methods have multilevel biases in their identifications due to both experimental and computational limitations (e.g. calling more deletions than insertions). A key aspect of our approach is that we calibrate all our calls against synthetic data sets generated from simulations of high-throughput sequencing (with realistic error models). This allows us to calculate sensitivity and the positive predictive value under different parameter-value scenarios and for different classes of events (e.g. long deletions *vs*. short insertions). We run our calculations on representative data from the 1000 Genomes Project. Coupling the observed numbers of events on chromosome 1 with the calibrations gleaned from the simulations (for different length events) allows us to construct a relatively unbiased estimate for the total number of SVs in the human genome across a wide range of length scales. We estimate in particular that an individual genome contains ~670,000 indels/SVs.

**Conclusions:**

Compared with the existing read-depth and read-pair approaches for SV identification, our method can pinpoint the exact breakpoints of SV events, reveal the actual sequence content of insertions, and cover the whole size spectrum for deletions. Moreover, with the advent of the third-generation sequencing technologies that produce longer reads, we expect our method to be even more useful.

## Background

One important goal in genomics is to determine the genetic differences among individuals and to understand their relationships to the phenotypic differences within a species, such as human beings. These variations consist of single nucleotide polymorphisms (SNPs) and structural variations (SVs) including short insertions/deletions (indels) and other more complex ones such as duplications and translocations. Because of the efficiency of genotyping methods and the central role they play in the genome-wide association studies, SNPs are currently the best catalogued and studied human genetic variations. Ubiquitous 1-bp indels, expansions of simple repeats and chromosomal anomalies have long been observed and acknowledged as the genetic bases for some human diseases [1,2]. Except for these old discoveries, however, indels and SVs have been much less studied due to their wide size range, the multitude in their types, and the lack of an efficient genotyping method. After several recent studies, however, their genetic significance starts to be appreciated: not only do they exist in large numbers in the human populations, they may also have a more significant impact on phenotypic variation than SNPs [3-7].

The microarray technology, array CGH, has been widely used to detect copy number variants (CNVs), a type of SV, with kilo-bases resolutions [5,8-11]. The advancement in high throughput sequencing technologies has enabled a new set of comparative approaches for CNV calling, such as the read-depth analysis [12-15], which computes the read coverage of different genomic regions, the read pair analysis, which focuses on cases where the distance between the two ends of a reads deviates more than expected when they are mapped back to the reference [4,16-18]. Accompanying the advancement of these experimental approaches, different computational methods for SV detection and their breakpoint refinement have also been developed [18-25].

**Figure 1:**
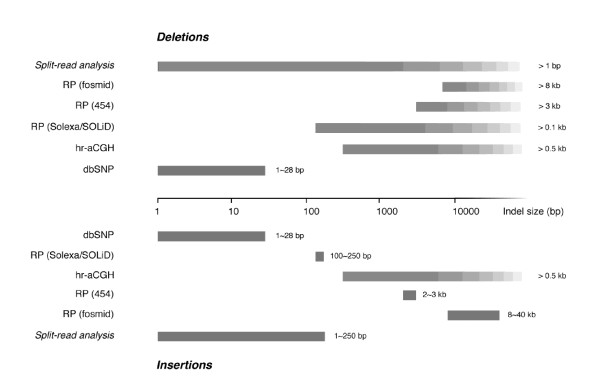
**The size spectrum of SVs identifiable to different methods**. No method can identify SVs of all different sizes. The black bars indicate the size ranges of discoverable SVs by different methods, which include the dbSNP database, the high-resolution array CGH (hr-aCGH), the read-pair (RP) method with fosmid, 454, and Solexa sequencing, and the split-read analysis. The range of detectable indels by RP depends on three values: the mean and the standard deviation between the distances of mapped read pairs and the multiple coefficient of s.d. for significance. These triple values are (40 kb, 2.8 kb, 3), (1 kb, 0.8 kb, 3), (250 bp, 25 bp, 6) for fosmid, 454, and Solexa sequencing, respectively.

Because indels/SVs come in various sizes, there is an additional aspect--the size coverage--to their detection. The aforementioned methods only partially address all the requirements of indel/SV detection to various degrees. For sequence insertions and deletions, indels/SVs are conventionally defined as micro-SVs of 1-10 bp and large ones over 1 kb, respectively. In the following text, wherever the context is clear we use SV as the encompassing term, subsuming small indels. Due to methodological limitations, SVs of middle lengths have only been minimally, if not at all, studied. Indeed, over the full spectrum of the SV size, only several small size spans are covered by current methods (Figure 1). Moreover, SV detection approaches described above (e.g. array/read-pair/read-depth based methods) cannot accurately locate the breakpoints of the SV events, nor can they reveal the actual sequence content of insertions. Such information can only be gained via the direct analysis of the read sequences, instead of based on the statistics of the mappings of such reads.

Here we report the split-read analysis, a sequence-based method that detects SVs through direct analysis of the mapping information of how high-throughput sequencing reads are aligned to the reference genome. Using alignment of read sequences to reference genomes with gaps, the method allows the precise identification of SVs covered by such reads. Building our method directly upon BLAT, a well-established sequence alignment program, we take advantage of the speed and the sensitivity of this popular sequence-to-genome alignment tool. However, more importantly, by considering both the sequencing and mapping errors in our assessment strategy to score each initial SV call, our method also takes into account the sequencing error model (especially for next-generation sequencing technologies, which were not generally available a few years ago), and distinguishes the different confidence levels in detecting different SVs based on the characteristics of supporting reads. Compared with the read-depth and the read-pair analyses, our sequence-based method can not only pinpoint the breakpoints of SV events, but also reveal the actual sequence content of insertions. The split-read analysis has another advantage--it can cover the whole size spectrum for deletions (Figure 1). We expect our method to be more useful in the future as the sequence reads become longer.

Due to both experimental and computational limitations, there are biases on multiple levels in the call sets generated by all current SV identification methods. In addition to their significantly more restricted size range of identifiable insertions than that of deletions, all current SV identification methods are sensitive to SVs of different length (Figure 1), and as a result studies using them have reported different numbers of SVs. One study using the read-pair method reported 241 SVs over 8 kb in a sampled genome [7], while another using the same approach but with a different molecular construct reported 422 and 753 SVs over 3 kb in two tested genomes [4]. In a study of whole-genome sequencing and assembly, 835,926 indels were identified in a diploid human genome [26]. Currently it is not known how many SVs, small or large, are in an individual human genome. Using empirical error models estimated from sequencing experiments to simulate high-throughput sequencing reads, we could not only parameterize our split-read method, but also, more importantly, quantify both false positive and false negative rates. Knowing these error rates enables us to estimate the total number of SVs of a given length in a human genome.

## Results

We have developed the split-read identification, calibrated (SRiC), a sequence-based method for detecting structural variants (SVs). It maps reads to the reference genome with gapped alignment and scores these mappings with consideration for sequencing and alignment errors. SRiC pinpoints exact SV breakpoints, reveals the sequence content of insertions, and covers the whole size spectrum for deletions. Simulation is used to calibrate SRiC, allowing unbiased estimation of the sensitivity and proportion of SVs across different length-scales.

### Analysis of the simulated sequence data

For sequencing simulations, instead of using the whole human genome, we use the diploid human chromosome 22 (NCBI36 assembly), which counts for 1% of the human genome but has a repeat content and a gene density both representative of the whole genome, to save computational processing time. To keep the local sequence environment of indels as found in a genome, we use indels identified in Venter's genome [26] in our sequencing simulation (Additional file 1).

#### Determining thresholds used in the analysis

Three thresholds are used in our split-read analysis: *t*_r_, the threshold on the ratio of the score of the best alignment to that of the second best as a measure of the uniqueness of the read, *t*_n_, the threshold on the number of supportive reads for 1-bp SVs, and *t*_c_, the threshold on the maximum centeredness (the maximum ratio of the smaller length to the bigger one of two flanking alignments of a read, Additional file 1, Figure S1) for large SVs.

**Figure 2:**
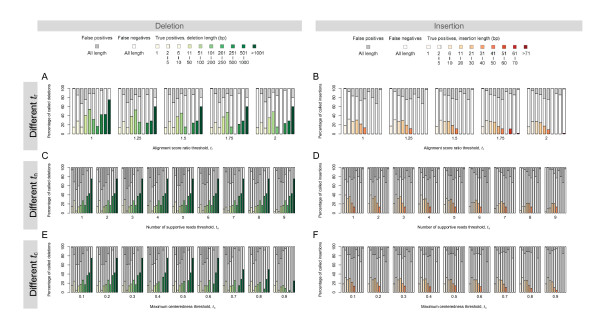
**Effect of different thresholds on SV identification**. Different sets of indels are called at combinations of different values for thresholds *t*_r_, *t*_n_, and *t*_c_. Each bar shows the percentages of the true positives, the false negatives, and the false positives of each call set, which are represented by the colored, the white, and the gray portions, respectively. The bars in different shades of green and red are used for the true positive calls of deletion and insertion of different length. (A-B) The alignment score ratio threshold, *t*_r_. SV are calls for a set of simulated reads using different *t*_r _while *t*_n _= 5 and *t*_c _= 0.1 are kept unchanged. (C-D) The number of supportive read threshold, *t*_n_. SV are calls for the same set of simulated reads using different *t*_n _while *t*_r _= 1 and *t*_c _= 0.1 are kept unchanged. (E-F) The maximum centeredness threshold, *t*_c_. SV are calls for the same set of simulated reads using different *t*_c _while *t*_n _= 5 and *t*_r _= 1 are kept unchanged.

To determine the score ratio threshold *t*_r _for the alignment preprocessing, we simulate ~5× sequence coverage that gives ~0.6 million 454 single-end 400-bp reads and then identify SVs using different values for the score ratio threshold *t*_r _(= 1.0, 1.25, 1.5, 1.75, and 2.0) while keeping the other two parameters fixed (*t*_n _= 5, *t*_c _= 0.1). The percentage of true positives, false negatives, and false positives of deletions and insertions identified at different *t*_r _values are plotted in Figure 2A-B. There is a small decrease in the number of identified SVs when *t*_r _is increased from 1.0 to 1.25. The further increases in *t*_r _from 1.25 to 2.0 only cause negligible changes to the SV identification results. Over all, the SR method is not very sensitive to *t*_r _when it is in the range of 1.0 and 2.0. This insensitivity is a result of unique mapping to the reference genome of most 454 reads, which are much longer than those produced by other next-generation sequencing technologies.

Two thresholds, *t*_n _and *t*_c_, are used for the initial SV calls (Inequalities 1 and 2). We vary the value of one of these two thresholds while fix the other to determine how they affect the accuracy and the sensitivity of the split-read method. Using the simulated sequence set with the ~5× coverage, we make SV calls with *t*_n _= 1, 2, ..., 9 while *t*_c _= 0.1 and *t*_c _= 0.1, 0.2, ..., 0.9 while *t*_n _= 5, count the true positive and the false positive calls, and calculate the percentage of true positives, false negatives, and false positives at each threshold combination. The results of this performance analysis as depicted in Figure 2C-F make it clear the effects that theses two thresholds have on the SV identification show a dichotomous dependency on the SV length. While *t*_n _affects the identification of short SVs, *t*_c _biases that of longer ones. In practice, we use the sequencing depth for *t*_n _(with a lower bound *n*_min _= 2) and set *t*_c _to 0.1. It is also clear that the method has different sensitivities in the size range of indels that it can detect: it is less sensitive to 1-bp indels because 454 sequencing is prone to over- or under-call bases in homopolymers and thus more a stringent threshold is needed to lower the number of 1-bp false positives.

#### Assessing how the read length affects the performance

**Figure 3:**
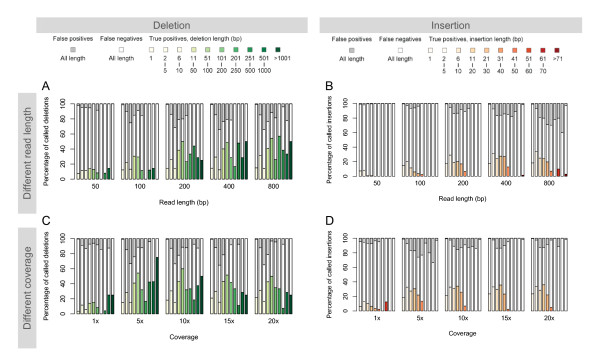
**Effect of sequencing on SV identification**. The lengths of the colored, the white, and the gray portions of each bar signify the percentages of the true positives, the false negatives, and the false positives, respectively. The bars in different shades of green and red are used for the true positive calls of deletion and insertion of different length. (A-B) Different read length. SV are calls for sets of simulated reads of different lengths with the same coverage (5×). (C-D) Different coverage. SV are calls for sets of simulated reads of the same lengths (~400-bp) with different coverage.

We first assess how the read length affects the SV identification by simulating single-end reads of 50, 100, 200, 400, and 800 bp long. For each read length, we generate sequences with ~5× coverage and analyze five sequence sets with the same set of method parameters (*t*_r _= 1, *t*_n _= 5, *t*_c _= 0.1). We compare the true SVs and the ones that we identified using the split-read analysis. The numbers of true and false positives of deletions and insertions identified using reads of different lengths are plotted in Figure 3A-B.

The general trend, which is expected and depicted in the figure, is that the SV identification is improved with longer reads. With 50-bp reads, the SV identification is the worst with low sensitivity for both short and long deletions. Because the length of discoverable insertions is capped by the read length, it is not surprising that at this read length none of the insertions of 20-bp and longer are found. When the read length is increased to 200 bp and longer, the sensitivity and the positive predictive value almost double for longer SVs. For deletions, 200-, 400-, and 800-bp reads seems to give comparable performance, and longer reads only bring marginal improvements to the results. The choice of read length for insertions identification is, however, a rather open-end question, as longer reads will always enable better identification of longer insertions.

#### Assessing the effects of sequence coverage on SV calls

We first simulate ~20× sequence coverage that gives ~2.5 million 454 single-end 400-bp reads. To assess how the sequencing depth affects the SV calls by the split-read analysis, we also simulate ~1×, 5×, 10×, and 15× sequencing coverage by down-sampling the 20× sequence set with appropriate numbers of reads (Table 1). We then identify SVs using default parameters (*t*_r _= 1.0, *t*_n _= coverage, *t*_c _= 0.1). The numbers of true and false positives of deletions and insertions identified at different sequencing coverage are plotted in Figure 3C-D. The general trend is that SV identification is improved with higher coverage but with diminishing returns. Comparing to the low coverage at 1×, there is a marked improvement to SV identification at higher coverage.

**Table 1:** Number of sequences in simulated and down-sampled datasets

Sequence type	Coverage	Number of sequences	Number of base pairs
Generated sequences	20×	2,477,629	994,491,814
Mapped sequences	20×	2,476,347	993,977,159
Used sequences	20×	2,476,088	993,873,276
	15×	1,857,784	745,693,367
	10×	1,236,929	496,489,303
	5×	619,052	248,478,809
	1×	123,633	49,625,857

Notes:

1. We use 49,691,432 bp as the size of human chromosome 22 for calculating the sequence coverage.

2. For 1~15× target sequence coverage, we sample from the used sequences in the 20×-coverage dataset.

**Figure 4:**
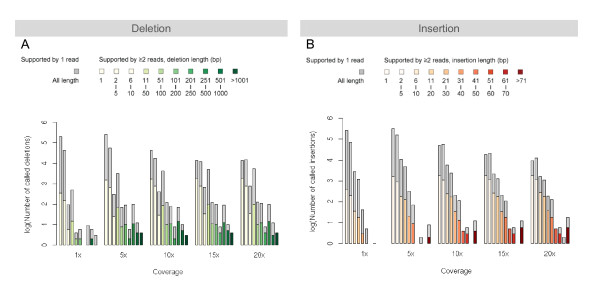
**Discoverable simulated SVs**. Not all SVs are identifiable, as some of them are not covered by any or enough sequence reads. The lengths of the gray and the colored portions of each bar signify the log-number of indels covered by only one and more than one read, respectively. The bars in different shades of green and red are used for the true positive calls of deletion and insertion of different length. A missing bar indicates a zero count. The counts of simulated deletions (A) and insertions (B) that are covered by at least two reads and by only one read are plotted as colored and gray bars.

To assess how sequencing coverage affects the sensitivity of our method, we determine the maximum sensitivity achievable in each simulated sequence set. The number of 'seeable' true SVs is affected by several factors, including the sequencing depth, the read mapping quality/uniqueness, and the minimum number of supportive reads required for an SV call. After the initial alignment processing to remove the mapping ambiguity, we count the number of supportive reads for the true SVs of different lengths and plot the number of true SVs with one and two or more supportive reads at different sequencing depth (Figure 4).

The sequencing depth has the most significant effect on short SVs. At 1× coverage, ~1,000 of true 1-bp deletions and insertions are supported by at least one read. When the coverage is increased to 2×, these numbers almost are doubled. As the coverage increases, the percentage of supported true SVs also increases but with a diminishing pace. 80~90% true SVs are supported by at least one read at 5× to 20× coverage. One supportive read is the absolutely minimum requirement for an SV call. To reduce the false positives, we require at least two supportive reads for every SV call. This global threshold has a much more significant effect on the low-coverage sequence set than on the high-coverage one: while the percentage of true deletions with two or more supportive reads is about the same as that of true deletions with one supportive read at 1× coverage, there are very few true SVs with only one supportive read at 10× or higher coverage.

### Performance assessment

Several different approaches have been used to extensively evaluate the performance of our SRiC method (Additional file 1). First, we compare SRiC with Pindel, the only published method that can detect SV breakpoints on the nucleotide level. The comparison between the numbers of SVs that these two methods can find in simulated datasets with the same SV placements shows SRiC has a significantly higher sensitivity than Pindel at every length simulated, whether it is of deletions or insertions (Additional file 1, Tables S1 and S2). Second, we apply our split-read analysis to 454 genomic reads generated for two individuals (CEU NA12878 sequenced to 0.5× and YRI NA19240 to 5×) and calculate the positive predictive values at different thresholds on the number of supportive reads after validating deletion calls using two experimental methods, respectively--array capture followed by sequencing and trio-array comparative genomic hybridization (CGH). The experimental result shows for the type of SVs under consideration SRiC can achieve 70-80% call accuracy (Tables 2 and 3).

**Table 2:** Array capture validation of SR called deletions. ^1, 2^

***t***_**n **_^**3**^	2	3	4	5	6	7
True positive	30	20	15	13	9	7
Positive	115	33	22	18	12	10
Positive predictive value ^4^	0.26	0.60	0.68	0.72	0.75	0.70

Notes:

1. 0.5× 454 reads for a CEU individual (NA12878) from the 1000 Genomes Project are used to make the SR deletion calls.

2. Only deletions longer than 500 bp are selected for array capture validation.

*3. t*_n_, is threshold on the number of supportive reads.

4. Positive predictive value = True positive/Positive.

**Table 3:** Trio-array CGH validation of result. ^1, 2^

***t***_**n **_^**3**^	2	3	4	5	6	7
True positive	48	23	17	12	9	7
Positive	76	27	20	15	11	9
Positive predictive value ^4^	0.63	0.85	0.85	0.80	0.82	0.78

Notes:

1. Only deletions longer than 50 bp called for an YRI individual (NA19240) from the 1000 Genomes Project are selected (randomly) for the trio-array CGH validation. Due to the data usage restriction, only validation results for deletions in chromosome 1 are used.

2. Not every validation test yields definite result. Inconclusive results are excluded from this table.

3. *t*_n_, threshold on the number of supportive reads.

4. Positive predictive value = True positive/Positive.

### Analysis of the 1000 Genomes Project data

A major sequencing project, the 1000 Genomes Project, has been launched to resequence the genomes of at least a thousand people from around the world using the new sequencing technologies to produce the most detailed map of human genetic variation for disease studies. As a proof of concept, we apply our split-read analysis to a set of 454 sequence reads generated by the 1000 Genomes Project for one individual.

The genome of an individual (NA19240) from the Yoruba in Ibadan, Nigeria has been sequenced using the 454 single-end method to ~5× sequence coverage. The sequencing generated ~49 million sequence reads, of ~17.6×10^9 ^bp in total. Mapping of the sequence reads (with a median length of ~400 bp) took ~136 hours of the wall time with exclusive access to 50 Dell PowerEdge 1955 nodes (each containing 2 dual core 3.0 Ghz Xeon 64 bit EM64T Intel CPUs model 5160 and 16 GB RAM) of a Linux cluster. Applying our SR method, we identify 13,426 deletions ranging from 1 bp to ~700 kb and 11,539 insertions ranging from 1 bp to 200 bp on the chromosome 1. Compared with 494 validated insertions in chromosome 1 from dbSNP (v129), 301 in both sets are at exactly the same genomic locations, which indicates a sensitivity of ~60% for validated insertions in dbSNP. This defines a lower bound on sensitivity as different genomic DNA sources are involved. The simulation used to compare the numbers of insertion and deletion calls (see above) enables us to determine the positive predictive values and the sensitivities of our SR method for indels identified in a sequence set at 5× coverage and subsequently estimate using equation (4) the total numbers of deletions and insertions of lengths in continuous ranges separately on chromosome 1 (Table 4). We estimate there are 53,431 SVs in chromosome 1 and extrapolate to 665,684 SVs in the whole genome of this individual.

**Table 4:** Corrected counts of SVs in the chromosome 1 and the whole genome of a Yoruba individual. ^1^

SV type	SV size range (bp)									
Deletion	1-5	6-10	11-50	51-100	101-200	201-250	251-500	501-1000	> 1000	Total
	
Chromosome 1	20,229	3,018	1,619	183	224	156	419	37	101	25,986
Whole genome ^2^	252,028	37,600	20,171	2,280	2,791	1,944	5,220	461	1,258	323,753
Insertion	1-5	6-10	11-20	21-30	> 30	Total				
					
Chromosome 1	22,187	3,743	1,074	228	213	27,445				
Whole genome	276,422	46,633	13,381	2,841	2,654	341,931				

Notes:

1. The true number of large SVs will be underestimated. However, this will have only a marginal effect the magnitude of the estimation of the total number of SVs in a large chromosome or in the whole genome.

2. The true number of SVs in the whole genome is estimated by extrapolation of the corrected number of SVs in chromosome 1 by the fold increase in size from chromosome 1 to the whole genome.

## Discussion

### Mapping reads to the reference genome

The size of the deletions covered by the split-reads can range up to tens of thousands of bases, and this makes BLAT well suited for mapping such reads back to the genome, since it not only allows small gaps and mismatches within the alignment like many other alignment tools, but also takes into account large gaps due to its initial purpose to handle introns in RNA/DNA alignments [27]. In short, unlike the alignment results from tools such as BLAST which will generate two distinct partial alignments for a split-read covering a large deletion event, the alignment results of BLAT can directly reveal the deletion event and its up- and down-stream alignments at the same time. Recently a new algorithm, Burrows-Wheeler Aligner's Smith-Waterman Alignment (BWA-SW), has been designed and implemented to align with gaps long reads such as 454 reads (~200 bp or longer) to the reference genome with higher accuracy and a faster speed than BLAT [28]. However, BLAT should be used to align 454 paired-end reads, because currently the average 454 read length is less than 400 bp and thus, the majority of sequences on both ends will be shorter than 200 bp.

For the non-split reads, however, using BLAT would be unnecessarily time-consuming, because their alignment results would usually only contain (if any) a small number of mismatches. Bowtie, a recently developed alignment tool, incorporates the Burrows-Wheeler transform technique to index and search the genome in a fast and memory-efficient manner, and is an immediate candidate for processing such reads [29].

The two-tiered alignment cascade is used to expedite the step of aligning reads to the reference genome. The first assortment step effectively fractions the sequence reads into two subsets: ones that can be uniquely mapped and ones that cannot. By limiting the gapped alignment of the reads in the former subset to their associated chromosomes, the tiered mapping approach removes the unnecessary mapping attempts and thus speeds up the alignment step. The speed gain is clearly related to the size ratio of the two read subsets: the more uniquely mappable reads, the bigger the speed gain. Because it is assessed by their 35-bp end tags, the genomic uniqueness of the reads is limited to the unique mappability of the 35-mers to the human genome. It has been estimated that 79.6% of the genome is uniquely mappable using 30-bp sequence tags. Since the human genome consists of 24 chromosomes, it is natural to use them as the bins for end tag assortment. It is, however, conceivable to fraction the human genome into large (e.g., 100 Mb) fragments with small (e.g., 1 kb) overlaps and use them as the assortment bins to further restrict the search space of the subsequent BLAT genomic mapping of the reads whose end tags are uniquely mapped.

### Parameterization of the split-read analysis

Five parameters are intrinsic to our split-read analysis alone: the alignment score ratio threshold *t*_r_, the threshold on the number of supportive reads for 1-bp SVs *t*_n_, the threshold on the maximum centeredness for large SVs *t*_c_, the minimum number of supportive reads for every SV identification *n*_min_, and the exponential decay parameter *λ*. For sequence reads that are mapped to multiple genomic locations, we use *t*_r _to control on what level of distinctiveness such reads can be used for the SV identification. A higher value of *t*_r _lowers the overall mapping ambiguity and thus reduces the number of false positives. This will, however, disqualify more correct alignments and in turn increase the number of false negatives. Small and large false SV calls have different origins: the former result from sequencing errors that under- or over-call bases while the later are mostly generated by misalignments. To count for such distinct error origins, two different threshold functions, separately parameterized with *t*_n _and *t*_c _using the same exponential base function, are used to make SV calls. *λ *controls how fast the threshold changes between 1-bp and large SVs and it is set to 1 in all of our split-read analyses. We require that there should be at least two supportive reads for every SV identified regardless of its length. This global threshold (*n*_min _= 2) dramatically reduces the false positive SV calls.

## Conclusions

Directly building our method upon BLAT, we take advantage of the speed and the sensitivity of this popular sequence-to-genome alignment tool. However, more importantly, we designed an assessment strategy to score each initial indel/SV call that takes into account both the sequencing and mapping errors. Compared with the existing read-depth and read-pair analyses, our sequence-based method can pinpoint the exact breakpoints of indel/SV events, reveal the actual sequence content of insertions, and cover the whole size spectrum for deletions. We thoroughly benchmarked and validated our SRiC method against the best available methods for detecting structural variants at relevant resolutions by using several different approaches to extensively evaluate the performance of our method. We illustrate the characteristics of our split-read method by applying it to both synthetic and experimental data sets. With the advent of the third-generation sequencing technologies that produce longer reads, we believe the split-read approach presented here can make a significant contribution to the study of indels/SVs.

## Methods

**Figure 5:**
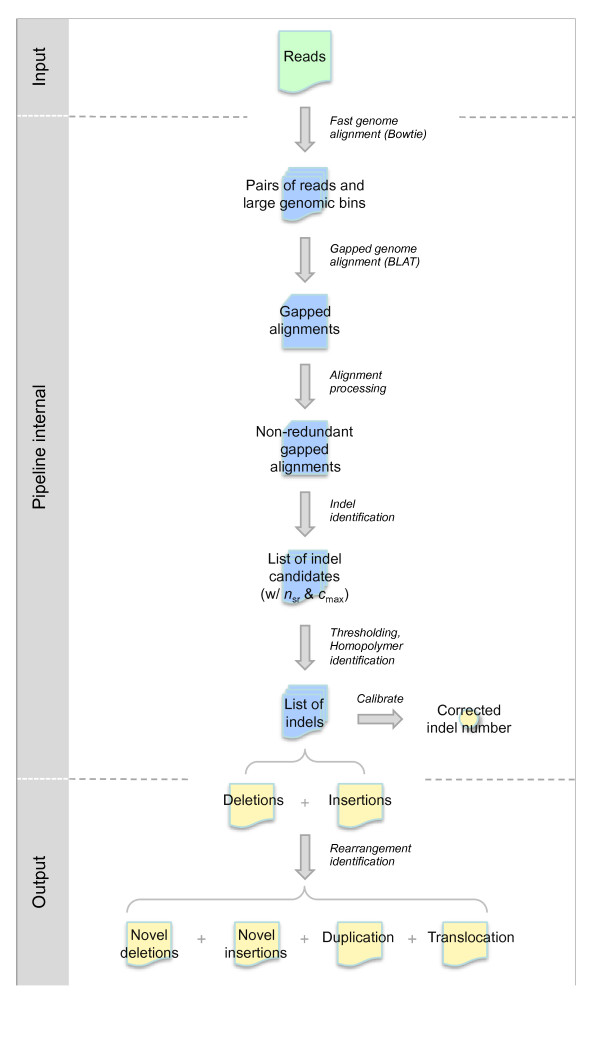
**The flowchart of the split-read analysis pipeline**.

Sequence data are analyzed in a stepwise fashion, as depicted in Figure 5. Below we describe our split-read analysis in detail.

### Data input

The data input for the split-read analysis are genomic read sequences. For sufficient alignability, these reads should have a length of hundreds of bases and currently can be generated by the Sanger sequencing or, to a much higher throughput, the 454 sequencing. However, we expect reads from other sequencing platform (e.g., paired Solexa reads with overlap) may also be used after preprocessing. The current system implementation only supports the widely used FASTA sequence format.

### Tiered sequence alignment

The sequence reads are first processed to remove any terminal ambiguous bases (Ns) and then mapped to the human reference genome (NCBI Build 36.1, UCSC hg18) using BLAT with parameters tuned for short sequences with maximum sensitivity (-*stepSize *= 5, -*tileSize *= 11, -*repMatch *= 10^6^, and -*fine*). Certain parts of the reference genome (such as low complexity regions and simple sequence repeats) can be masked out by replacing the sequences with Ns to disallow indel identification in these regions. When the set of reads is large, the aforementioned direct approach to sequence mapping will be very time-consuming. To enhance the speed of the alignment step, we use a tiered approach instead by dividing our alignment process into two steps: a fast initial assortment of the reads followed by a complete gapped alignment.

Briefly, we first take 35-mer tags at each end of a read, map them to the whole reference genome using Bowtie, a rapid alignment tool for short reads, look for those end tags that can be mapped uniquely to the genome, and assort the corresponding reads by their associated chromosomes. Using BLAT to obtain the gapped alignments, we then align the assorted reads only to their targeted chromosomes and the remaining reads whose ends cannot be uniquely mapped to the whole genome. Thanks to the modularity of the implementation, Bowtie and BLAT used here can be replaced by other alignment tools, such as MAQ and BLAST, with minor modifications.

For all uniquely mappable reads, this tiered mapping approach can speed up the alignment to the human genome by 24 times on average. The whole process is parallelized, and for a total of ~3 million reads (~60 GB in size) it takes less than an hour to finish the assortment step with 80 CPUs of a computer cluster. On average, ~70% of the single-end reads of a sequenced individual could be assorted by the aforementioned algorithm. As a result, we anticipate an overall enhancement of the alignment speed by 3 folds.

### Alignment preprocessing

If a read is mapped to the genome uniquely, we keep its alignment without additional requirements. Otherwise, its alignments are scored and the alignment ratios calculated. The alignments are then sorted on their scores, ratios, and the number of alignment blocks. We only keep the top alignment when its score is at least *t*_r _times (to be determined by simulation) as big as that of the second best on the sorted list. Moreover, DNA amplification as part of the library preparation procedure increases the likelihood that a DNA fragment is sequenced multiple times. Redundant sequence reads (the same chromosome, the same strand, and the same start position) generated from the same DNA fragments are removed to prevent the inflation of the count of reads that are supportive of SVs.

For paired-end sequence reads, they are processed to release the end sequences with the pairing information preserved for later use after the linker sequence is identified and removed. The end sequences are then mapped and processed like the single-end reads as described above. Because of restriction on how two ends are mapped relatively to each other on the genome, the pairing information increases the accuracy of their genomic placement. To avoid excessive assumptions on the distribution of the insert length, we make the minimum requirement that two ends of a read should be mapped to the same strand of the same chromosome. Only read ends that make unique concordant pairs are used in the downstream analyses.

### Insertion/deletion and rearrangement identification

**Figure 6:**
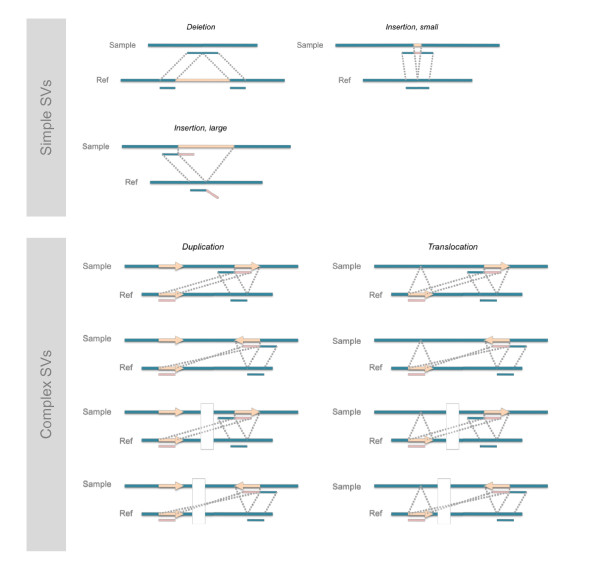
**The conceptual diagrams of the split-read analysis**. SVs can be detected by sequence reads spanning their break points. The split-read analysis can directly identify deletions, small insertions, and the boundaries of large insertions. After the identification of SVs, duplications and translocations can be isolated out based on matching of insertions and deletions. Breakages in blue genomic lines denote different chromosomes.

After sequence alignment and placing reads at their most likely locations in the reference genome, the split-read analysis searches these locations for insertions and deletions in the sample genome by identifying reads that encompass SV break points (Figure 6). To find deletions in the sample genome, we search for reads that when aligned to the reference genome split on the same strand of a chromosome. Even though a deletion of an arbitrary size can be detected as long as it is covered by one or more reads, the size of insertions that can be directly detected in full is limited by the read length. To find small insertions that are fully included in the reads, we search for reads whose terminal sequences can be aligned next to each other on the reference genome. For large insertions, we look for their boundaries, which are found in reads that, except one of their ends, can be aligned to the reference genome continuously in one block.

For each identified SV, we count the number of reads that 'support' it, *n*_sr_, and measure its centeredness in each supportive read, *c_i _*(*i *= 1, ..., *n*_sr_), the ratio of the smaller length of its two flanking alignments to the bigger one. It is easy to see that 0 <*c_i _*≤ 1 and if there are multiple supportive reads for an SV it is the maximum centeredness that matters the most (because the evidence best supportive of presence is the most informative). Thus, each SV identification is associated with two scoring quantities: the number of supportive reads, *n*_sr_, and the maximum centeredness, *c*_max _(Additional file 1, Figure S1).

Considering the lists of deletions and (small) insertions together in conjunction with each other, we resolve their final SV identities as novel deletions, novel insertions, duplications, and translocations. To do this, we first extract from reads the sequences of insertion that are at least 20 bp long and then align them to the reference genome using BLAT. An insertion is classified as 'novel,' if it cannot be aligned perfectly without gaps. Otherwise, it is a duplication and potentially a translocation. To be the latter, at least one location of the perfect alignments to the reference genome needs to be precisely covered by a read with deletion. The novel deletions are the whole set of deletions excluding those 'used' by translocations.

### SV call set obtention through SV call filtering and sequencing error identification

Sequencing errors or spurious sequence alignments can both lead to SVs calls by the split-read analysis. The majority of such false positives can be removed by imposing a simple global threshold that requires every SV to be found in at least two nonredundant reads. We further refine the call list, and since the false positives of the short and the long SVs arise from distinct sequencing and alignment errors, respectively, we treat the short and the long SV calls differently.

**Figure 7:**
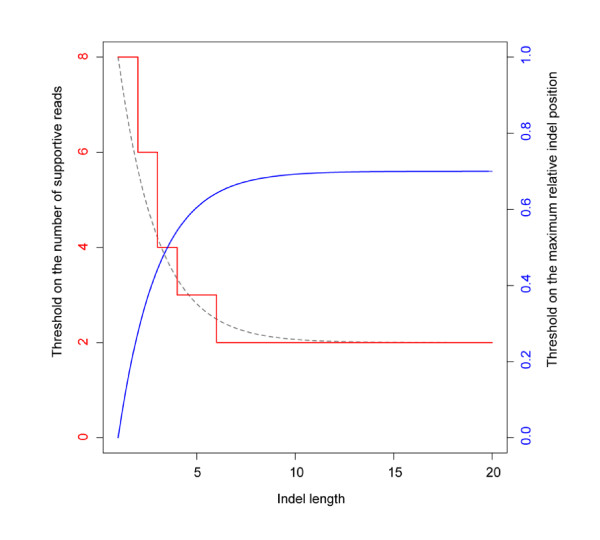
**The curves of the threshold functions**. Each SV call is scored by the number of supportive reads and the maximum centeredness in those reads. The thresholds on these two quantities are determined by two threshold functions, plotted as the read and the blue curves, respectively. The gray dashed curve is the threshold function for the number of supportive reads before rounding. The parameter values used for the shown functional curves are *λ *= 1, *t*_n _= 8, and *t*_c _= 0.7.

Based on the SV length, false SV calls have different origins: small sequencing errors, large misalignments, and a mixture in between. Sequencing errors that under- or over-call bases manifest as deletions and insertions in the sequence reads when they are aligned to the reference genome. False SVs of this origin have the characteristics that they are very short, mainly 1-bp SVs, and also occur largely in a random fashion. In contrast, false large SV calls are mostly generated by misalignments in which the SVs are located very closely to one end of the reads. False SV calls with lengths in the narrow middle range are thought to be a mixture of errors from either of the origins. We use exponential functions to model such a dichotomy and the quick transition between small and large SV lengths. Given their distinct origins, we remove false small and large SV calls by requiring *n*_sr _and *c*_max _to meet the following two conditions simultaneously:(1)(2)

in which *t*_n _is the threshold on the number of supportive reads for 1-bp SVs, *t*_c _the threshold on the maximum centeredness for large SVs, *l *the length of the SV in base pair, *n*_min _the minimum number of supportive reads for every SV identification (effectively the threshold on the number of supportive reads for large SVs), and *λ *the exponential decay parameter that controls how fast the threshold changes between 1-bp and large SVs (Figure 7). *n*_min _= 2 and λ = 1 are used in all of our split-read analyses.

The error characteristics of different sequencing platforms are approximated by different error models. The simplest model, which considers only 1-bp SVs, specifies the probability, *p*_e_, of 1-bp SVs due to sequencing errors. After the initial call filtering, we perform a significant test for each 1-bp SV, where the null hypothesis is that the SV probability is the same as the probability specified by the error model. Because *p*_e _stays the same for all sequence reads that contain the same 1-bp SV, we use the binomial distribution to calculate the *P*-value, which is the probability of seeing the same number and more of the reads having this SV out of the total number of reads covering this site, *n*_cr_, given the SV probability from the error model, *p*_e_:(3)

After the Bonferroni correction for multiple tests, the null hypothesis is rejected if *P *< 0.01.

Because of the increased likelihood of both under- and over-calling bases in homopolymers by 454 sequencing technology, for each SV that is a part of a homopolymer we perform a significant test after the initial call filtering, where the null hypothesis is that the SV probability is the same as the probability specified by the error model (Additional file 1, Figure S2). The calculation of the *P*-value is described above.

### Calibration of the number of SVs in a genomic region

Previous steps will produce a set of SV calls for the assayed genomic region. Because the performance of our SR method can be assessed and quantified with the positive predictive value and the sensitivity by extensive simulation, we can use these error rates to derive less biased estimate of the number of SVs in that genomic region.

Given the number of SVs identified in sequence reads covering a genomic region (e.g., a chromosome or indeed the whole genome) to a certain depth, the total number of SVs of a certain length can be estimated using the positive predictive value and the sensitivity determined in a simulation data set with the same sequencing coverage:(4)

in which , *PPV_l, c_*, and *S_l, c _*are the number of SVs, the positive predictive value, and the sensitivity for SVs of length *l *(bp) observed in reads giving *c*-x sequence coverage. This method is not applicable to SVs of a certain length that are not observed (i.e., ). For large SVs, it is more sensible to use a range of length, instead of discrete lengths.

## Authors' contributions

ZDZ implemented the method, performed the analyses, and drafted the manuscript. JD, HL, and AA helped with the implementation of the method. AEU and MS participated in the array-CGH validation for the 1000 Genomes Project. MG conceived of the study and helped to draft the manuscript. All authors read and approved the final manuscript.

## Supplementary Material

Additional file 1 **Supplementary materials**. PDF file includes additional Methods and associated references, Tables S1 and S2, and Figures S1, S2, and S3. Click here for file
