# Supplementary material for: Identification of genomic indels and structural variations using split reads
Source: BMC Genomics. 2011 Jul 25;12:375. doi: 10.1186/1471-2164-12-375 (PMC3161018; doi:10.1186/1471-2164-12-375)
Supplement: Additional file 1 — Supplementary materials. PDF file includes additional Methods and associated references, Tables S1 and S2, and Figures S1, S2, and S3. [file 1471-2164-12-375-S1.PDF]

SUPPLEMENTARY MATERIALS

FOR

Identification of genomic indels and structural variations using split reads

Zhengdong D. Zhang [1, §], Jiang Du [2], Hugo Lam [3], Alex Abyzov [1], Alexander E. Urban [4], Michael Snyder [5], Mark Gerstein[1, 2, 3, §]

[1] Department of Genetics, Albert Einstein College of Medicine, Bronx, NY 10461, USA
[2] Department of Computer Science, Yale University, New Haven, CT 06520, USA
[3] Interdepartmental Program in Computational Biology and Bioinformatics, Yale University, New Haven, CT 06520, USA
[4] Department of Psychiatry and Behavioral Sciences, Stanford University, Stanford, CA 94305, USA
[5] Department of Genetics, Stanford University, Stanford, CA 94305, USA

[§] Corresponding authors (E-mails: zhengdong.zhang@einstein.yu.edu, mark.gerstein@yale.edu)

Keywords: insertion, deletion, structure variation,
split read, high-throughput sequencing

+

Considerations specific to the 454 sequencing

Currently we focus on finding SVs in sequence reads generated by the 454 sequencing, as it is the only widely used high-throughput sequencing technology that produces long sequence reads (~500 bp). In our split-read analysis, there are processing steps specific to the 454 sequencing error characteristics. Other high-throughput sequencing technologies can soon produce sequence reads long enough for our analysis. The error characteristics of those sequencing methods can be specifically modeled and dealt with in a similar manner.

We perform simulations of 454 read sequences to enable the evaluation of the false positive rate of the split-read analysis. The simulation proceeds through the following steps. First, an underlying genomic sequence—either a whole genome or a part of it (i.e., a chromosome)—from which the read sequences are drawn, is selected, and the SVs of specified lengths and counts are randomly generated in it. Second, the SV-loaded genomic sequence is sheared by randomly picking DNA fragments with lengths sampled from a lognormal distribution with its mean and standard deviation (7.8 and 0.29 respectively, both in log-space) estimated from a typical 454 sequencing run in the 1000 Genomes Project and then uniformly placing fragments along the sequence. Third, sequence read are generated by taking sequences of either end of DNA fragments with lengths set to a particular value (e.g., 200 bp) or sampled from an empirical length distribution of sequence reads produced by a typical 454 run (currently using the Roche GS-FLX-Titanium technology, with an average read length ~400 bp calculated from real sequence reads). Finally, the constructed reads are then subject to simulation of 454 sequencing errors (see below).

To reproduce typical 454 sequencing errors, we apply an error model in our simulation that generates the background nucleotide insertions and the homopolymer SVs, the two major causes of errors in 454 sequencing. In the error model, we assume that signals observed from a homopolymer of length *n* follow a normal distribution, $N\left(\mu=0.2,\sigma^2=0.1^2\right)$, while the background follows a lognormal distribution, $\log\text{-}N\left(\mu=-1.72,\sigma^2=0.47^2\right)$ (Figure S2) (Korbel, Abyzov, Mu, Carriero, Cayting, Zhang, Snyder and Gerstein 2009). We use intersection points of the curves as cutoff points for calling a particular DNA sequence for a given signal. For instance, signals in the range 0.56 to 1.43 are called as a single sequenced nucleotide, rather than a homodimer. Nucleotide flow is simulated in the following order: T, A, C, G. For every nucleotide sequenced (including homopolymers and single nucleotides), the observed signal is generated either from a background distribution—in cases where the flowed nucleotide was different from the nucleotide to be sequenced—or otherwise from the corresponding normal distribution. The overall sequencing error rate is 2.5%.

Performance assessment

Comparison with Pindel

Currently Pindel (Ye, Schulz, Long, Apweiler and Ning 2009) is the only published method that can detect indel breakpoints on the nucleotide level. Using a method different from our approach, it identifies the breakpoints of large deletions (1bp-10kb) and medium-sized insertions (1-20bp) from 36 bp paired-end short reads. For performance appraisal, we compare the numbers of indels that Pindel and our split-read analysis can find in simulated datasets with the same indel placements. We first simulate the following datasets:

- Indel sets: we randomly disperse 100 of each of 1-, 2-, 5-, 10-, 100-, 300-, 500-, 1000-, 5000-, 10000-bp heterozygous deletions and 1-, 2-, 5-, 10-, 25-, 50-, 100-, 150-, 200-, 250-bp heterozygous insertions in the reference human chromosome 22.
- Sequence set for SR: we simulate 454 single-end reads to ~5x coverage from the above indel-added chromosome 22 with a length distribution similar to that of sequence reads produced by a typical 454 run.
- Sequence set for Pindel:
    - With the same <u>coverage</u> as the set for SR: we simulate 36-pb paired-end reads to ~5x coverage from the above indel-added chromosome 22.
    - With the same <u>sequencing cost</u> as the set for SR: assuming 454 sequencing is 10 times more expensive than Solexa sequencing (Du, Bjornson, Zhang, Kong, Snyder and Gerstein 2009), we simulate 36-pb paired-end reads to ~50x coverage from the above indel-added chromosome 22.

We process the simulated datasets as follows:

- 5x 454 single-end reads: the split-read analysis with parameters $t_r = 2$, $t_n = 5$, $t_c = 0.1$.
- 5x and 50x 36-bp paired-end reads: Pindel with default parameters.

In Table S1 and S2, we list the number of true positive and false positive deletion and insertion calls made by SR and Pindel. From these tables, we make the following several observations:

- At 5x sequence coverage, Pindel can find only a few simulated indels.
- Compared at every length simulated, whether it is of deletions or insertions, SR shows a significantly higher sensitivity than Pindel.
- Due to predisposition of 454 sequencing to indel errors in homopolymers, SR has a low positive predictive value for small indel calls. Pindel has a consistent high PPV across its indel calls.

Compared to Pindel, in addition to higher sensitivity, our SR method can also identify insertion/deletion double events in the resequenced sample genome.

Experimental validation of SR deletion calls

<u>Validation using array capture followed by sequencing</u>. SR calls of deletions longer than 500 bp for a CEU individual (NA12878) with 0.5x 454 sequencing coverage are validated using array capture hybridization and sequencing. DNA from the flanking neighborhood of predicted deletion breakpoints are captured on a customer-designed microarray and re-sequenced using massively parallel sequencing. (For details of this method see Urban et al., in preparation.) See

Table 2 for the validation result of the SRiC deletion calls using array capture followed by sequencing.

Validation using trio-array CGH. SR calls of deletions longer than 50 bp for a YRI individual (NA19240) with 5x 454 sequencing coverage are validated using high-resolution CGH arrays, which contain 42 million probes and have been recently used in CNV discovery and genotyping by the Genome Structural Variation Consortium(Conrad, Pinto, Redon, Feuk, Gokcumen, Zhang, Aerts, Andrews, Barnes, Campbell et al. 2009). Using these arrays, the probe intensities of the two individuals are compared to a common reference individual (NA18051). The distribution of log-ratio probe intensities in a predicted deleted region is compared with the null distribution. Statistically significant deviation from the null validates the deletion. Additionally, the distribution is also compared with empirical distributions of probe intensities from regions known to have 2:2, 2:1, and 1:2 copy number ratios. This allows arbitration between alternative models (2:2 vs. 2:1, 2:2 vs. 1:2) and, if reaching statistical significance, validation/invalidation of a deletion call. Validation result is inconclusive for all calls that cannot be validated by either approach or invalidated by the latter one (alternative models). See Table 3 for the validation result of the SRiC deletion calls using the trio-array CGH.

Comparing the numbers of insertion and deletion calls

We compare the efficiency of calling deletions and insertions up to 30 bp by the SR analysis. Long SVs are excluded from the comparison, because insertions longer than the read length are known to be undetectable and ones whose lengths are on a par with the read length can only be detected with a diminished efficiency. The low detectability of long insertions is a result of the requirement that sequences of insertions need to be encapsulated in sequence reads for their full detection.

To make the comparison simple, we simulate the same number of small insertions and deletions: we first randomly disperse 100 of each of 1-, 2-, 3-, ..., 30-bp deletions and insertions in the reference human chromosome 22 and then generate 20 sequence sets, each with ~5x coverage. All sequence sets are analyzed with the same method parameters ($t_r = 2$, $t_n = 5$, $t_c = 0.1$). The numbers of identified deletions and insertions of different lengths are plotted in Figure S3. The box plot shows that except 1-bp and 2-bp SVs there are more deletions than insertions identified at a given SV length and the discrepancy becomes more pronounced as the SV length increases.

Deletions and insertions of genomic sequences are not detected with an equal efficiency. Identification of insertions is more difficult than that of deletions in two aspects: there are more false negatives of both large and small insertions and more false positives of micro insertions. As Figure 1 shows, depending on what insert length is used, the read pair approach can detect deletions of >4 kb or >8 kb but insertions of only 2-3 bp or 8-40 bp. From the figure, it is also clear that the split-read analysis can cover the whole size spectrum for deletions but is limited to 1-250 bp for insertions using the 454 Titanium reads (the longest among reads

generated by the next-generation sequencing technologies). The much smaller coverage of insertions than deletions is a direct consequence of the limitation on the physical size of the sequence constructs or the sequence reads, both of which cannot be arbitrarily large to accommodate the inserted sequences. The direct comparison of the split-read calls for small insertion and deletion shows that except 1-bp and 2-bp SVs there are more deletions than insertions identified at a given SV length (Figure 7). This bias for deletions reflects the fact that it is easier for a sequence mapping program such as BLAT to open gaps in the long genomic sequences than in the short sequence reads (Figure 2). The difficulty in detecting insertions also stems from more false positives of micro insertions. There is a small (but nevertheless existing) probability for 1-bp insertions in the background (the gray under-curve area in the inset of Figure S2). Also, for a given length of homopolymer, it is more likely for a 1-bp overcall than a 1-bp undercall. For example, given an arbitrary base, it is more likely to be called as a duplet than a null (the blue under-curve area is bigger that that of the green area in the inset of Figure S2).

References

Conrad, D.F., D. Pinto, R. Redon, L. Feuk, O. Gokcumen, Y. Zhang, J. Aerts, T.D. Andrews, C. Barnes, P. Campbell et al. 2009. Origins and functional impact of copy number variation in the human genome. *Nature*.

Du, J., R.D. Bjornson, Z.D. Zhang, Y. Kong, M. Snyder, and M.B. Gerstein. 2009. Integrating sequencing technologies in personal genomics: optimal low cost reconstruction of structural variants. *PLoS Comput Biol* **5:** e1000432.

Korbel, J.O., A. Abyzov, X.J. Mu, N. Carriero, P. Cayting, Z. Zhang, M. Snyder, and M.B. Gerstein. 2009. PEMer: a computational framework with simulation-based error models for inferring genomic structural variants from massive paired-end sequencing data. *Genome Biol* **10:** R23.

Ye, K., M.H. Schulz, Q. Long, R. Apweiler, and Z. Ning. 2009. Pindel: a pattern growth approach to detect break points of large deletions and medium sized insertions from paired-end short reads. *Bioinformatics* **25:** 2865-2871.

SUPPLEMENTARY TABLES

Table S1. Simulated deletions called by the split-read analysis and Pindel. [1, 2]

Simulated deletions:										
Size (bp)	1	2	5	10	100	300	500	1000	5000	10000
Number	100	100	100	100	100	100	100	100	100	100
SR deletion calls on 5x read set:										
TP	15	27	46	44	76	69	59	65	57	66
FP	5	14	30	20	2	2	0	0	0	0

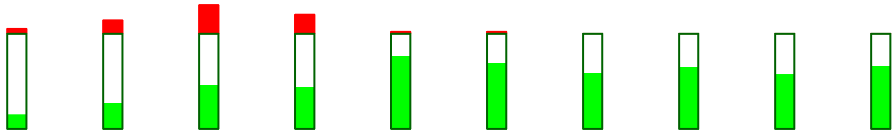

Pindel deletion calls on 5x read set:										
TP	0	0	0	0	0	0	0	0	2	0
FP	0	0	0	0	0	0	0	0	0	0

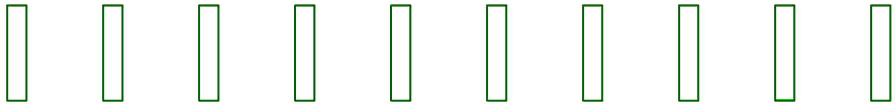

Pindel deletion calls on 50x read set:										
TP	0	0	10	15	10	12	7	14	14	12
FP	0	0	0	0	0	0	0	0	0	0

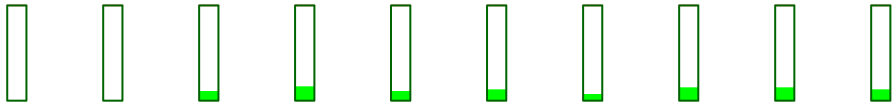

Notes:

1. The true positive (TP) and false positive (FP) calls are identified by comparing the called deletions with the simulated ones with the requirement that the overlap is reciprocally over 50%.
2. Of the graphic presentation of the numbers of the calls, the height of the box with a green border is the number of true deletions, which is 100 in every case, the green bar is the number of TPs, and the red bar is the number of FPs.

Table S2. Simulated insertions called by the split-read analysis and Pindel. [1]

Simulated insertions:										
Size (bp)	1	2	5	10	25	50	100	150	200	250
Number	100	100	100	100	100	100	100	100	100	100
SR insertion calls on 5x read set:										
TP	9	32	61	50	35	29	23	22	5	2
FP	42	14	13	4	3	2	0	3	0	0
	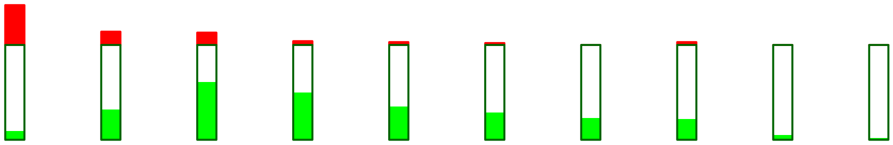									
Pindel insertion calls on 5x read set:										
TP	0	0	2	0	0	0	0	0	0	0
FP	0	0	0	1	0	0	0	0	0	0
	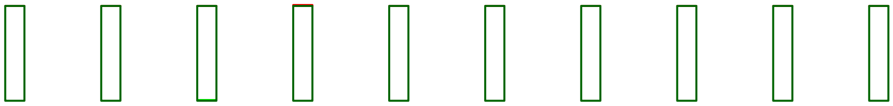									
Pindel insertion calls on 50x read set:										
TP	0	0	31	32	0	0	0	0	0	0
FP	0	0	3	4	0	0	0	0	0	0
	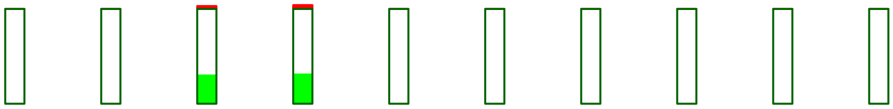									

Notes:

1. The true positive (TP) and false positive (FP) calls are identified by comparing the called insertions with the simulated ones with the requirement that the overlap is reciprocally over 50%.
2. Of the graphic presentation of the numbers of the calls, the height of the box with a green border is the number of true insertions, which is 100 in every case, the green bar is the number of TPs, and the red bar is the number of FPs.

SUPPLEMENTARY FIGURE LEGENDS

Figure S1. Scoring quantities of identified SVs. Each SV identification is associated with two scoring quantities: the number of supportive reads, n_{sr}, and the maximum centeredness, c_{max}

Figure S2. Distribution of signal frequencies for homopolymers of different length in 454-sequencing data. Signal distributions are given for the background (the gray curve), for single nucleotides ('homopolymer' size of 1 bp, the black curve), and for homolpolymers of sizes 2 to 10 bp. Inset: Over- and under-calls around one base pair. Extremely large and small sequencing signal of a homopolymer will cause over- and under-calls of the homopolymer. The gray and blue areas are the probabilities of 0-to-1 bp and 1-to-2 bp over-calls, while the green and red areas are the probabilities of 1-to-0 bp and 2-to-1 bp under-calls.

Figure S3. Box plots of numbers of calls for deletion and insertion of different lengths. 100 deletions and insertions of each length are generated in all 20 simulated sequence sets. The numbers of deletions and insertions of each length called for 20 sets are plotted to show the median (horizontal line dissecting box), the upper and lower quartiles (top and bottom box edges), the highest and lowest data values that fall within 1.5 times the interquartile distance from the box edges (lines extending to 'whiskers'), and data outliers outside of this range (circles).

SUPPLEMENTARY FIGURES

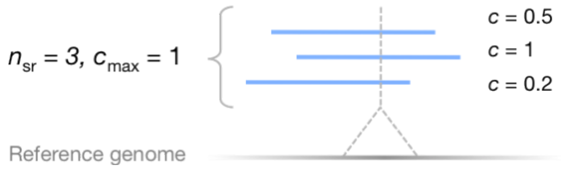

Figure S1.

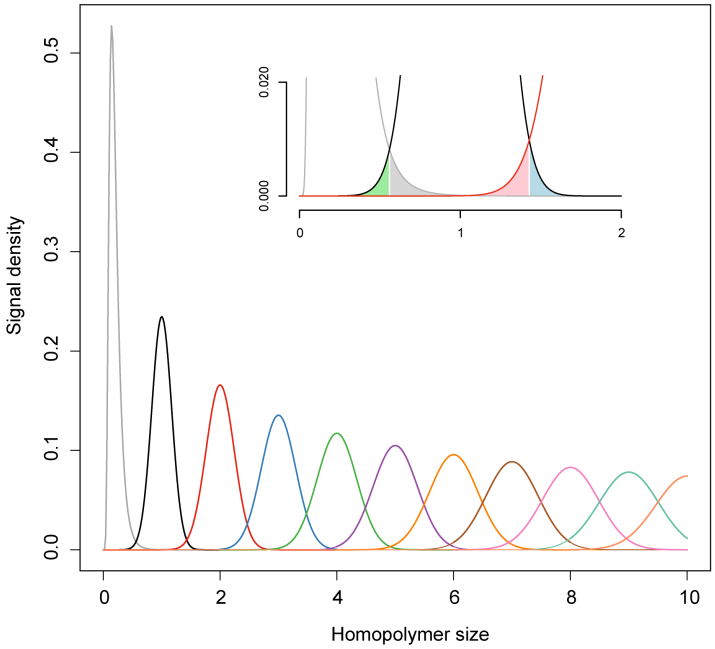

Signal density
Homopolymer size
0.0
0.1
0.2
0.3
0.4
0.5
0
2
4
6
8
10
0.000
0.020
0
1
2

Figure S2.

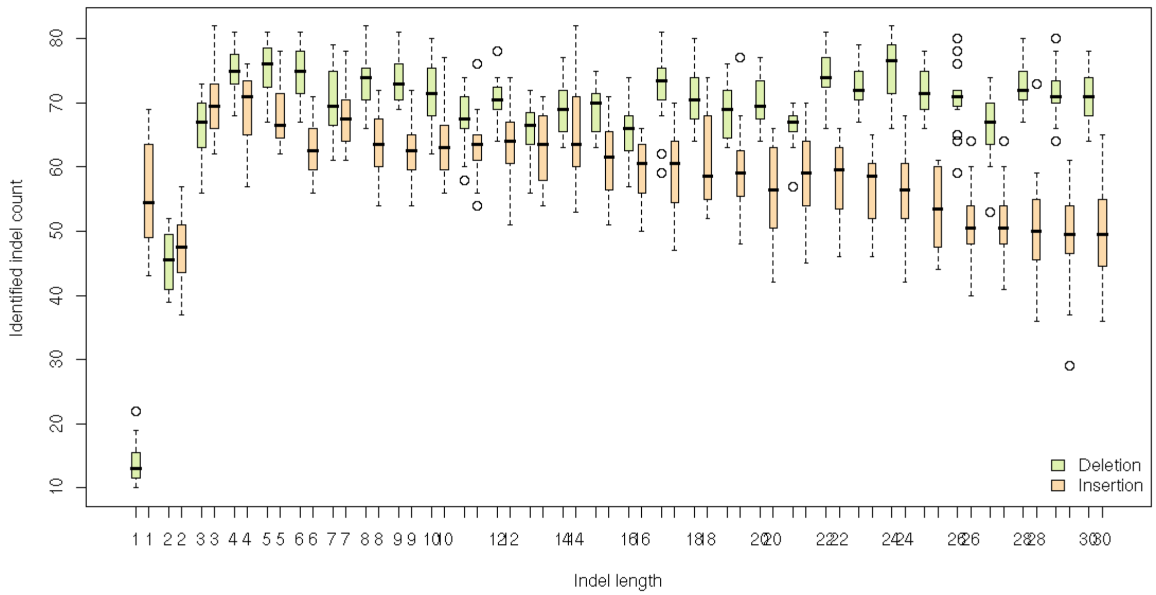

Figure S3.
